# Management of Cerebral Cavernous Malformations: From Diagnosis to Treatment

**DOI:** 10.1155/2015/808314

**Published:** 2015-01-05

**Authors:** Nikolaos Mouchtouris, Nohra Chalouhi, Ameet Chitale, Robert M. Starke, Stavropoula I. Tjoumakaris, Robert H. Rosenwasser, Pascal M. Jabbour

**Affiliations:** ^1^Department of Neurological Surgery, Thomas Jefferson University and Jefferson Hospital for Neuroscience, Philadelphia, PA 19107, USA; ^2^Department of Neurological Surgery, University of Virginia School of Medicine, Charlottesville, VA 22908, USA; ^3^Division of Neurovascular Surgery and Endovascular Neurosurgery, Department of Neurological Surgery, Thomas Jefferson University Hospital, 901 Walnut Street, 3rd Floor, Philadelphia, PA 19107, USA

## Abstract

Cerebral cavernous malformations are the most common vascular malformations and can be found in many locations in the brain. If left untreated, cavernomas may lead to intracerebral hemorrhage, seizures, focal neurological deficits, or headaches. As they are angiographically occult, their diagnosis relies on various MR imaging techniques, which detect different characteristics of the lesions as well as aiding in planning the surgical treatment. The clinical presentation and the location of the lesion are the most important factors involved in determining the optimal course of treatment of cavernomas. We concisely review the literature and discuss the advantages and limitations of each of the three available methods of treatment—microsurgical resection, stereotactic radiosurgery, and conservative management—depending on the lesion characteristics.

## 1. Introduction

Cerebral cavernous malformations (CMs), also known as cavernomas, are vascular abnormalities of the brain that are comprised of clusters of abnormal, hyalinized capillaries surrounded by hemosiderin deposits and a gliotic margin [1–3]. The vasculature is filled with blood and is thrombosed in varying degrees.

The incidence of cerebral CMs ranges from 0.4% to 0.8% in the general population, but they are the most common vascular abnormality, making up 10–25% of all vascular malformations. They can be found in several locations in the brain, but 70–80% of them are supratentorial [1, 4]. Supratentorial CMs most frequently present with new-onset seizures, but headaches are also common, while infratentorial CMs usually lead to progressive neurological deficits [5, 6]. Intracranial hemorrhages of varying severity can also occur in both supratentorial and infratentorial lesions. The annual risk of hemorrhage is 0.7%–1.1% per lesion in patients with no history of hemorrhage but rises to 4.5% in patients with a previous intracerebral hemorrhage (ICH) [7–10]. The risk of rupture also depends on the location of the lesion, its size, the presence of a developmental venous anomaly (DVA), and the patient's gender. Superficial CMs have a lower ICH risk than the deeply located ones. More specifically, the ICH risk for infratentorial CMs is 3.8% but 0.4% for supratentorial CMs [9, 11]. Additionally, female patients have a worse prognosis than male patients [12, 13].

Approximately 40–60% of patients with CMs have the familial form, inherited in an autosomal dominant pattern due to a heterozygous mutation in one of the three genes, CCM1, CCM2, and CCM3, found on the 7q, 7p, and 3p chromosomes, respectively [1]. The familial form usually results in multiple cavernomas, whereas the sporadic disease typically leads to a single cavernoma [14, 15]. The products of the CM genes have been shown to play a major role in angiogenesis by associating with cytoskeletal and interendothelial cell junction proteins in neural tissue [2]. Loss-of-function mutations in one of these genes disrupt the endothelial cell-cell junctions, leading to extensive vascular abnormalities and increased permeability.

**Figure 1:**
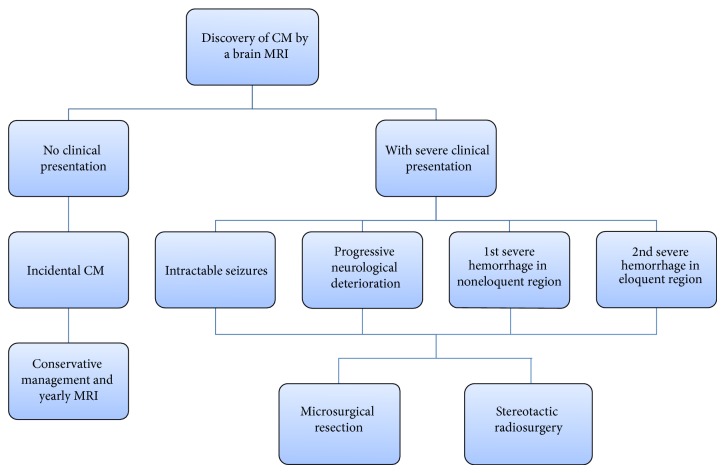
Flow-chart of the work-up and management of patients with cavernous malformations. Once a cavernomas is diagnosed via an MRI of the brain, deciding the course of treatment depends on the clinical presentation of the patient. Purely incidental cavernomas are managed conservatively and followed by yearly MRI scans. Cavernomas are treated by microsurgical resection or stereotactic radiosurgery if the patient is experiencing severe symptoms, such as intractable seizures, progressive neurological deterioration, one severe hemorrhage in a noneloquent region of the brain, or at least two severe hemorrhages in eloquent brain. Selecting between resection and radiosurgery depends on the location of the lesion and the severity of the presentation as explained in this paper.

Microsurgical resection, stereotactic radiosurgery, and conservative management are the three methods of treatment for CM lesions. Deciding how to manage a CM patient depends on a multitude of factors that are discussed in this paper (Figure 1). While there are many studies on each of these methods, the natural history of CM lesions is complicated and not clearly understood, which may potentially compromise the conclusions drawn regarding the efficacy of the treatment used. Temporal clustering of hemorrhages has been shown in patients with untreated CMs; a 2.4-fold decrease (*P* < 0.001) in the rate of bleeding was observed 2.5 years after the first hemorrhage [16]. Some are more skeptical regarding temporal clustering due to the absence of a second period of increased risk within 5 years of follow-up [17]. In that case, the risk of hemorrhage is naturally reduced 2 to 3 years after a hemorrhagic event.

## 2. Clinical Imaging Used for CM Management

The diagnosis of cavernomas is more difficult than other vascular diseases since CMs are angiographically occult malformations. Angiography is only able to detect the existence of abnormal venous drainage associated with CMs; thus other imaging techniques are needed to provide an accurate diagnosis. Conventional T1- and T2-weighted MR imaging, gradient echo sequences, high-field MRI, susceptibility-weighted imaging, diffusion tensor imaging, and functional MRI are some of the advanced techniques that are being used for diagnosis of CMs or for intraoperative navigation during the treatment of deeply located lesions.

### 2.1. Conventional T1- and T2-Weighted MR Imaging

Conventional MR imaging is able to accurately detect symptomatic cavernous malformations, which are surrounded by a ring of hypointensity due to hemosiderin deposits from recurring microhemorrhages [7, 18]. The CM lesions are divided into four types based on their appearance on MR imaging. Type I lesions appear hyperintense on T1- and T2-weighted imaging due to a hemosiderin core from subacute hemorrhage. Type II lesions contain loculated hemorrhages enveloped by gliotic tissue, presenting as a mixed signal on both T1 and T2 sequences. On T2 imaging, type II lesions also have a hypointense rim, resulting in the “popcorn” appearance [19]. Type III lesions are diagnosed by the presence of an isointense core, indicating chronic resolved hemorrhage, typically seen in familial CM. Type IV lesions are small malformations that can only be seen in gradient recalled echo (GRE) MRI as hypointense foci and are thought to be capillary telangiectasias [18, 20].

### 2.2. Gradient Recalled Echo (GRE) MR Imaging

GRE MR imaging is a key method for diagnosis of CMs due to its ability to display hemosiderin-filled brain tissue with a very distinct hypointensity. Studies on familial CMs have shown that conventional MR imaging detected an average of 5 lesions per patient, while T2-weighted GRE MRI identified an average of 16 lesions per patient [21]. GRE MRI not only is more capable of identifying all of the lesions present, but also delineates the lesions more precisely [22]. While it has several benefits, it is important to note that GRE MRI augments the apparent size of the CM lesion. Additionally, GRE MR images may show multifocal lesions in elderly patients with hypertension and a history of stroke, but they must not be mistaken for familial CMs. They result from hypertensive angiopathy and are located in periventricular areas [7].

### 2.3. Use of High-Field MRI for Diagnosis of CMs

The use of conventional 1.5 T MR imaging is limited, as CM lesions may not be visualized unless high-field MR imaging is used. Using MR strengths of up to 14 Tesla, several studies have illustrated the ability of high-field imaging to visualize lesions as hypointensities that were not otherwise apparent [23–25]. Depending on the strength, lesions may appear to be larger than in reality. More specifically, high-field imaging at 7 T causes lesions to appear 11% larger than in conventional imaging techniques [25].

### 2.4. Susceptibility-Weighted MR Imaging

Susceptibility-weighted (SW) imaging is very advantageous for detecting CM lesions because it accurately recognizes deoxyhemoglobin and hemosiderin. It is also considered the only method capable of detecting unbled CM lesions and telangiectasias [7]. SW imaging has been shown to delineate CMs more precisely as well as detect additional CM lesions that cannot be seen by conventional imaging methods. De Souza et al. studied 15 patients with familial CMs and found 5.7, 26.3, and 45.6 lesions per patient using T2-weighted imaging, T2 ∗ GRE imaging, and SW imaging, respectively. SW imaging detected 1.7 times more lesions than T2 ∗ GRE (*P* = 0.001) [26]. Other studies on familial CMs corroborate these findings; however, SW imaging is not superior to T2 ∗ GRE imaging regarding the detection of sporadic, solitary CMs or clusters of CMs associated with a DVA [7]. Additionally, using sequential SW imaging with contrast agent may prove very useful in distinguishing venous vasculature from small regions of hemorrhage, but this application of SW imaging needs to be studied further [27].

### 2.5. Diffusion Tensor (DT) Imaging and fMRI Used Intraoperatively

DT and fMR imaging are used intraoperatively to better visualize the lesions and the surrounding parenchyma in order to improve the surgical outcome even if the lesions are deeply located in eloquent areas. DT tractography allows the surgeon to visualize the white matter tracts, which frequently cross through the hemosiderin rim of the CM lesion [19]. Several studies have shown the successful use of DT imaging in locating the tracts and avoiding them, significantly decreasing the morbidity associated with CM resections [28–30]. fMR imaging measures activity-dependent changes in cerebral blood flow, which becomes especially useful when resecting CM lesions located in eloquent brain [31, 32]. Zotta et al. show the use of fMRI for surgical planning and intraoperative navigation and report higher rates of completely seizure-free patients [33]. The use of fMRI neuronavigation enabled them to follow a more aggressive approach on the perilesional tissue without increasing the morbidity rate [33].

There is promising evidence supporting the use of DT and fMR imaging intraoperatively to achieve better outcomes without an increase in the morbidity and mortality rates. However, most studies on the neuronavigation techniques involve only a small number of patients; further investigation of these techniques is warranted using a larger number of patients to ensure generalizability.

## 3. Microsurgical Resection

Cavernous malformations are dynamic lesions that may exhibit enlargement, regression, or even de novo formation [3, 34]. They are resected after patients have experienced multiple hemorrhages in eloquent areas, or a single hemorrhage in a noneloquent area that is associated with deteriorating neurological deficits [35]. In addition, experiencing severe symptoms, such as cardiac or respiratory instability, and the presence of a CM lesion within 2 mm from the pial surface are important indications for surgery [5]. Patients are treated with steroids for 1 to 2 weeks prior to the surgery in order to limit the edema and allow for the CM resection. If there is a DVA associated with the CM lesion, its resection should be avoided because removing the DVA entails a high risk of venous infarction [9]. Moreover, upon excision of the CM lesion, gliosis, calcification, and hyaline degeneration often take place and may complicate the procedure [5].

Complete removal of the lesion is required in order to prevent recurring hemorrhagic events, but that depends on the neurosurgeon's experience [36]. Resection of the hemosiderin ring must also be accomplished if seizure surgery is performed. Rebleeding has been shown to occur in 40% of cavernoma remnants after surgery, which is why a postoperative MRI within 72 hours is strongly recommended. If remnants are found, surgical intervention is needed as early as possible [37].

The complication risks associated with surgical intervention vary with the location of the CM lesion. Amin-Hanjani et al. showed that the patients' overall neurological condition was good or excellent in 100% of patients with cranial nerve CMs, 97% of those with lobar CMs, 87.5% of those with cerebellar CMs, 75% of those with spinal cord CMs, and 64% of those with brainstem CMs [38].

### 3.1. Supratentorial Cavernomas

The vast majority of CM lesions are located supratentorially and most commonly present with seizures, focal neurological disorders, and headaches depending on their location. Surgical resection of symptomatic CM lesions located in noneloquent areas is always recommended, as it has been shown to be safe as well as effective in treating epilepsy and preventing future hemorrhages [4, 39]. However, deciding on whether to resect a CM becomes more complicated when the lesion is located in an eloquent area of the brain and is barely symptomatic or completely asymptomatic. The use of frameless stereotaxy and intraoperative fMR imaging significantly reduces the risk of complications and establishes microsurgical resection as a favorable treatment method for most supratentorial CM lesions. Gralla et al. report complete removal of the CM lesion using intraoperative navigation in all of the patients studied [40].

Surgical resection of CM lesions has also been shown to allow long-term seizure control with acceptable morbidity and mortality risks. Englot et al. studied 1,226 patients with supratentorial CM-related seizures and showed that 75% of them became seizure-free upon CM resection. They also identified that gross-total resection, surgery within the 1 year of symptom onset, CM size less than 1.5 cm, and having a single CM lesion are factors that significantly increase the rate of successful seizure control [41]. Additionally, Sommer et al. used intraoperative 1.5 T MRI (iopMRI) and neuronavigational software to surgically treat epilepsy in 26 patients. They managed complete seizure control in 80.8% of their patients, as observed during a 47.7-month mean follow-up period [42]. Using iopMRI was significant in completely removing the CM lesion in 23% of their patients, who would otherwise have a low chance of being seizure-free [41, 43]. Nonetheless, in spite of the promising data on the effectiveness of CM resection in treating epilepsy, antiepileptic drugs should still be the first-line treatment for CM-related epilepsy due to the complication risks associated with surgery.

### 3.2. Brainstem Cavernomas (BSCMs)

BSCMs make up approximately 20–35% of all CMs and are deeply located in the medulla, pons, and midbrain [1]. The annual hemorrhage risk (AHR) for spontaneous BSCMs has been shown to be 0.25–6.5% per patient-year, while the risk rises to 3.8–35% if the patient has a history of prior hemorrhage [14, 44, 45]. Some studies report the AHR to range from 4.5% up to 60% in patients with history of a prior hemorrhage [46]. Due to their location, hemorrhages from BSCMs exert pressure on the surrounding cranial nerve nuclei and tracts, leading to neurological deficits in 60% of the patients [47]. The blood is slowly absorbed and the symptoms often attenuate over time.

The resection of BSCM lesions entails a greater complication risk than resecting other CM lesions. This surgery has been shown to often produce symptoms that resemble a hemorrhage due to the increased pressure in the brainstem, but the symptoms disappear in the majority of the patients. Due to the increased complication risk, the main criteria for selecting surgery are severe clinical presentation, including hemorrhage, and location within 2 mm from pial surface. In case the lesion has severe clinical presentation but is deeply seated, surgery is selected only if the lesion is large and accessible [48, 49]. Frischer et al. resected BSCM lesions with a median volume of 2 cm^3^ when a microsurgical corridor was available [48].

There are many studies that have examined the short- and long-term effects of microsurgical resection on patients with BSCM lesions. More specifically, Li et al. reported complete resection of the CM lesion in 95% of the patients with 35.1% of the patients' condition worsening postoperatively [50]. After a mean 89.4-month follow-up period, only 10.3% of the patients remained in worse condition than preoperatively [50]. Frischer et al. achieved complete excision in 90% of patients and showed that 50% of the patients with residual lesions experienced additional hemorrhages, resulting in an AHR of 8.8% postoperatively [48, 51]. A study by Garrett and Spetzler on patients with BSCM lesions examined the neurological status of 137 patients immediately after the surgery and found that 72.3% of them had improved or were identical to their preoperative baseline [46]. After a mean follow-up period of 52 months, 89.2% of them had returned to their normal routine. In the same study, 88% of the patients who received surgery were the same or better than preoperatively. However, 3.5% of the patients died from a surgery-related cause. Additionally, 58% of their patients acquired new deficits, and 12% of all the patients treated had permanent deficits [46].

Surgical resection has been shown to be effective in treating BSCM lesions, yet some studies present more concerning results. Abla et al. showed that 7.7% of patients who underwent resection experienced a rehemorrhage postoperatively and 36% acquired permanent neurological deficits, and the surgery resolved some or all of the preoperative symptoms in only 45% of the patients [44]. This is illustrated by the change in the average Glasgow Outcome Score (GOS), which was 4.4 on admission, decreased to 4.2 at discharge, but rose to 4.6 at follow-up [44]. Ferroli et al. found that 44% of their patients acquired new neurological deficits postoperatively and 66% of whom eventually returned to or improved from the preoperative baseline, while the deficits were permanent in the rest of the patients [52].

The exact location of the lesion in the brainstem and the experience of the neurosurgeon are the key to limiting the risk of complications and the postoperative emergence of neurological deficits. A large variation in the findings of different studies is observed, which may be due to the varying number of patients in each study in addition to the variability in the experience of the surgeon and the advancement of technology.

## 4. Stereotactic Radiosurgery

While microsurgical resection is the standard treatment for cavernomas, the risk of complication is not negligible when treating deeply located, eloquent CMs. When the surgical risk is high, stereotactic radiosurgery (SRS) can be used to prevent the natural progression of the lesion.

Several studies have suggested that SRS is a safe and effective method for treating surgically inaccessible CMs [53] (Table 1). Lunsford et al. studied patients with high-risk CM lesions who received SRS treatment and showed that the risk of hemorrhage decreased from 32.5% to 10.8% in the first 2 years and to 1% after 2 years [54]. In the same study, 18.4% of the patients experienced adverse radiation effects; however the percentage decreased to 8% in the more recent patients as technology advanced [53, 54]. Lu et al. performed a meta-analysis study that included 178 patients with brainstem CMs and they showed a significant reduction in the AHR post-SRS treatment. According to their findings, the relative risk for hemorrhage was 0.161 (95% CI 0.052–0.493; *P* = 0.001), while 11.8% of the patients acquired transient or permanent neurological deficits [55]. Furthermore, Lee et al. studied the effectiveness of gamma knife radiosurgery (GKRS) on patients with brainstem cavernous malformations and showed that SRS should be considered as a treatment for brainstem CMs even in patients with only one previous hemorrhage [56]. The first study group consisted of patients who received GKRS treatment after a single hemorrhagic event, while the second study group had a history of 2 or more CM hemorrhages. The first group had an AHR of 7.06% within the first 2 years, and 2.03% after two years [56]. The second group had an AHR of 38.36% before SRS, 9.82% within the first two years post-SRS, and 1.50% after 2 years. However, 22.2% of the patients in the second group experienced new or aggravated neurological deficits from recurring hemorrhages [56]. In addition, Park and Hwang studied 21 patients who had experienced at least one hemorrhage due to their brainstem intra-axial CMs (1.55 hemorrhages per patient on average) [57]. They observed patients for a median of 32 months and noted that the risk of hemorrhage decreased from 39.5% to 8.2% after GKRS, while only one patient (5%) experienced adverse radiation effects. The risk of bleeding was reduced to 0% two years postoperatively [57].

Despite the growing evidence supporting SRS for inoperable CMs, some of the aforementioned studies present significant radiation-induced adverse effects and neurological deficits, as summarized in Table 1. It is thus imperative to note some limitations when studying the efficacy of SRS that may affect the SRS-associated morbidity rate. Hemorrhages due to cavernomas tend to occur in clusters with long intervals without any hemorrhages (temporal clustering) [16]. Barker II et al. showed that the incidence rate of a second hemorrhage within 1 year was 14%, but the cumulative incidence increased to 56% after 5 years [16]. Hence, the decreased risk of hemorrhage recurrence observed when following patients for an average 32 months could be due to the hemorrhage-free intervals seen in CM patients. Moreover, Poorthuis et al. conducted a metaregression analysis on CMs treated by SRS and showed that there is not any statistically significant association between the risk factors of each patient and the outcome of the procedure [58]. Their findings suggest that there is a large variation in the findings of SRS studies and that the long-term effects of SRS treatment still need to be determined [58].

## 5. Conservative Management

Due to the potential risks associated with interventional treatment, there have been several studies on the effectiveness of medical management of CMs, allowing lesions to progress naturally and only alleviating the clinical symptoms. Fernández et al. reported that surgical treatment of CM patients with nonrefractory epilepsy did not significantly decrease the risk of future seizures when compared with conservative management. They observed 17 patients who received medical management for 5 years and 12 of them (70.6%) remained seizure-free [59]. In contrast, other studies report that CM patients that received conservative treatment had a poorer outcome in the long term than patients who received surgical intervention (42% versus 9% resp.) [12, 36]. Garrett and Spetzler studied 14 patients who were managed conservatively and found that 50% of them improved or remained at their baseline, 29% became worse, and 7% died, while 14% did not complete the study [46].

While some positive findings on conservative management have been reported, there are important limitations to these studies. First of all, the number of patients studied is not large enough to represent the large range of cases seen in the hospital. Additionally, these studies were not randomized clinical trials, in which patients are randomly assigned to either surgical or medical management. Instead, researchers retrospectively studied patients who did not receive surgical treatment for various reasons, for example, because they maintained good control of their epilepsy, because of the location of CM, or simply because they declined surgery. However, this introduces bias to these studies, because it is very likely that these patients had less symptomatic lesions and therefore a milder and safer natural progression than the average CM patient. In addition, observing patients for only a few years is not sufficient, since the point of interventional management is to eradicate the risk of developing any permanent neurological deficits in the long term and prevent the AHR from increasing with time.

## 6. Conclusion

Cerebral cavernomas are the most common vascular abnormality, yet they are often undiagnosed. Using advanced imaging techniques such as T2 ∗ GRE sequences, high-field MR, and susceptibility-weighted imaging, we are now able to detect all of the CM lesions present in the brain. With the help of the intraoperative neuronavigational techniques, diffusion-tensor and fMR imaging, neurosurgeons can resect deep-seated lesions in eloquent areas of the brain with minimal new neurological deficits and low mortality and morbidity rates. Stereotactic radiosurgery has also advanced significantly and can be used to effectively treat inoperable cavernomas. More studies on SRS are needed though in order to examine its long-term effects on the neurological status of patients. Additionally, the natural history of cavernomas needs to be investigated further for it is crucial when assessing the efficacy of the methods of treatment.

## Figures and Tables

**Table 1:** Efficacy of treatments for brainstem cavernous malformations (BSCMs).

Treatment	Recurrence of hemorrhage		Permanent neurological deficits	Radiation-induced adverse effects
Microsurgical resection	0.4% [50] 7.7% [44] 8.8%^*^ [48]		10.8% [5] 12% [46] 15% [52] 36% [44]	N/A

	1st 2 years	After 2 years		

Stereotactic radiosurgery	7.06% [56] 8.2% [57] 8.22% [60] 10.8% [54] 14%^**^ [16]	0.6% [48] 1% [54] 1.37% [60] 1.50% [56] 2.03% [56]	7.3% [17] 22.2% [56]	4.1% [61] 5% [57] 8.2% [56] 11.8% [60] 8%–18.4% [54]

^*^Patients with residual lesions postoperatively.

^**^This refers to the rate of hemorrhage within the first year after SRS.
